# Environmental and genetic regulation of plant height in soybean

**DOI:** 10.1186/s12870-021-02836-7

**Published:** 2021-01-25

**Authors:** Qing Yang, Gaoming Lin, Huiyong Lv, Cunhu Wang, Yongqing Yang, Hong Liao

**Affiliations:** Root Biology Center, College of Resources and Environment, Fujian Agriculture and Forestry University, Fuzhou, 350002 China

**Keywords:** Shoot architecture, Plants height, Genotype, QTLs, Agro-meteorological factors, Soil properties

## Abstract

**Background:**

Shoot architecture is fundamentally crucial to crop growth and productivity. As a key component of shoot architecture, plant height is known to be controlled by both genetic and environmental factors, though specific details remain scarce.

**Results:**

In this study, 308 representative soybean lines from a core collection and 168 F_9_ soybean progeny were planted at distinct field sites. The results demonstrated the presence of significant genotype × environment interaction (G × E) effects on traits associated with plant height in a natural soybean population. In total, 19 loci containing 51 QTLs (quantitative trait locus) for plant height were identified across four environments, with 23, 13 and 15 being QTLs for SH (shoot height), SNN (stem node number) and AIL (average internode length), respectively. Significant LOD ranging from 2.50 to 16.46 explained 2.80–26.10% of phenotypic variation. Intriguingly, only two loci, ***Loc11*** and ***Loc19–1***, containing 20 QTLs, were simultaneously detected across all environments. Results from Pearson correlation analysis and PCA (principal component analysis) revealed that each of the five agro-meteorological factors and four soil properties significantly affected soybean plant height traits, and that the corresponding QTLs had additive effects. Among significant environmental factors, AD (average day-length), AMaT (average maximum temperature), pH, and AN (available nitrogen) had the largest impacts on soybean plant height. Therefore, in spite of uncontrollable agro-meteorological factors, soybean shoot architecture might be remolded through combined efforts to produce superior soybean genetic materials while also optimizing soil properties.

**Conclusions:**

Overall, the comprehensive set of relationships outlined herein among environment factors, soybean genotypes and QTLs in effects on plant height opens new avenues to explore in work aiming to increase soybean yield through improvements in shoot architecture.

**Supplementary Information:**

The online version contains supplementary material available at 10.1186/s12870-021-02836-7.

## Background

With the world population continually increasing, the demands placed upon agriculture to supply enough food will remain a great challenge for the foreseeable future [1]. Increasing crop yields has often been highlighted as a potential solution for meeting the challenge of feeding our growing population [2, 3]. Field-scale plant traits, such as plant density and lodging resistance, are critical determinants of grain yield for many crops. As such, ideal shoot architecture is considered one of the most important breeding targets for many crops [4, 5]. A key component of ideal shoot architecture is plant height. In rice, wheat and maize, shorter stem lengths contribute to higher yields through improved resistance to lodging [6–10]. For instance, the wide-spread incorporation of semi-dwarf cultivars into wheat and rice breeding programs throughout Asia in the 1960s and 1970s was an important factor in ushering the Green Revolution [11–13]. In soybean, advantageous shoot architectures are considered important components of numerous high yielding semi-dwarf cultivars, such as Hobbit87, Charleston and Apex [8, 9]. At present, the optimal height for current commercial soybean cultivars is typically 70–90 cm, with shorter or taller stands leading to yield reductions [14–17]. In short, existing evidence strongly suggests that ideal shoot architectures often depend on suitable plant heights, which, as yet, remains to be fully exploited for developing new high yielding cultivars.

As a characteristically quantitative trait, plant height displays significant variation among genetic backgrounds [18, 19]. Traditional breeding processes are time and labor consuming efforts, while, in modern breeding programs, marker-assisted selection (MAS), which has been successfully applied for many crops, allows for rapid selection of desirable traits [20]. Exploring more genetic resources in MAS efforts might, therefore, accelerate the process of breeding soybeans with suitable plant heights for optimal yields. To date, numerous QTLs associated with plant height have been identified in many crops, and several corresponding genes also have been identified through map-based cloning. Moreover, a portion of these genes have been proven to play critical roles in multiple breeding programs [7, 21]. For example, the Green Revolution gene, *sd1* (*semi-dwarf*), is associated with shortened rice plants, where it also improves lodging resistance [22]. Meanwhile, the well-known wheat dwarf gene, *Rht* (*reduced height*), which confers lodging resistance and increased allocation of assimilates to the grain, has been found in 70% of commercial wheat cultivars worldwide [21, 23]. Moreover, overexpressing *ZmPIN1a* (PIN-FORMED proteins) in maize significantly reduces plant height, internode length and ear height, all of which leads to shoot architectures that thrive in high-density cultivation conditions [24]. Finally, similar functions have been well documented for other QTLs or genes responsible for plant height, including *qDH1*, *qDTH8, D18* (*DWARF 18*), *D61* (*DWARF 61*), *brd1* (*brassinosteroid-dependent 1*), *HTD2* (*high tillering and dwarf 2*), *IPA1* (*ideal plant architecture1*), *MPH1* (*MYB-like gene of plant height 1*), *SLR1* (*slender rice1*), and *Sdd(t)* (*dominant semi-dwarf*) [7, 25–31]. In soybean, 239, 37 and 28 QTLs distributed across most of the 20 soybean chromosomes have been associated with plant height, stem node number and internode length, respectively, according to publicly available data (https://www.soybase.org). Among these QTLs, two loci, *Dt1* (*indeterminate growth 1*) and *Dt2* (*semideterminate growth 2*), have been well documented as associated with soybean shoot architecture and final grain yield. The existing evidence suggests that *dt1*(*determinate growth 1*) and *Dt2* act synergistically in regulating stem development through termination of apical growth, which leads to decreases in plant height and stem node number [20, 32–35].

Beyond genetic effects, crop shoot architecture is also influenced considerably by environmental conditions [36]. For example, elongation of the main stem may be promoted while the outgrowth of lateral buds is inhibited under low light intensity conditions [5]. Additionally, most plant physiological processes remain active only within the 0–40 °C temperature range. However, optimal temperatures vary among different physiological processes, with, for example, 18 °C being reported as the optimal night temperature for tomato stem elongation [37]. As a facultative short-day plant, soybean is influenced by both day-length and temperature, both of which also play critical roles in the formation of shoot architecture [38–40]. For example, soybean exposed to short photoperiods and high temperatures in low latitude regions typically exhibit early flowering, short periods of vegetative growth, short plant heights, and great reductions in yield [41, 42]. Meanwhile, several sensory loci or genes conferring sensitivity of shoot architecture to photoperiod have been identified and cloned from soybean, most notably *E1* - *E9* (*early flowering and maturity*) and *J* (*long-juvenile locus*) [43–49]. However, despite these numerous reports on the impacts of photoperiod on soybean shoot architecture, only few experiments have addressed temperature effects. Beyond meteorological factors, soil properties are also known to affect crop shoot architecture. As is widely known, crops require a suitable range of nutrients to meet the demands of growth and development. However, most agriculture soils cannot supply adequate quantities of all nutrients necessary to meet high yielding crop demands, so farmers continue to rely on fertilization. Evidence gathered to date suggests that the main fertilizer elements, N, P and K, might function in shoot architecture development. For instance, in cotton, wheat and rice, supplying N fertilizer leads to increases in plant height through formation of longer internode segments, but not increases in node number [7, 50, 51]. In contrast, K fertilization significantly reduces internode length [52, 53].

Soybean is a major source of oil and protein for food and feed [54, 55], though average yields globally are lower than obtained for other grain crops, such as rice, maize and wheat [2]. According to published data, in 2016, 81.3% of global soybean production was occurring in three countries in North and South America, including the United States, Brazil and Argentina. On the other hand, China is the largest consumer of soybeans, despite the fact that China only accounts for 3.57% of the global soybean production [56]. In China, the major soybean production areas include six disparate regions [57], each with soil properties that are distinct from the other regions. Most soils in the South China region belong to acidic soil types with low pH values and poor nutrient conditions, which is similar to soil conditions in Brazil and Argentina [41, 42, 56, 58]. Soil from the Huanghuaihai region and the lower-middle reaches of the Yangtze River basin tends to have higher pH values and more available nutrients than South China counterparts, which makes them similar to many soil types found across the USA [56, 59]. Despite these similarities between Chinese soils and soils found elsewhere, and in spite of Chinese farmers applying plentiful and, at times, excessive fertilizers in the field, average soybean yields in China (1.8 t ha^− 1^) are far lower than the average yields obtained in the USA (3.51 t ha^− 1^), Argentina (3.02 t ha^− 1^), or Brazil (2.91 t ha^− 1^) [56]. These situations imply that neither soil properties nor the amount of fertilizers applied are limiting factors for soybean yield in China. This suggests that fertilizer management, which is typically neglected by Chinese farmers and breeders, might be the critical factor for increasing soybean yields to levels in line with the yields reported from leading soybean producing countries. Moreover, excessive fertilization of soybean often leads to significant yield reductions, possibly due to development of poor shoot architectures producing taller and, more massive plants yielding less grain and growing less resistant to lodging [60]. Elucidating the effects of soil properties, particularly nutrient supplies, on shoot architecture development might, therefore, help breeders and farmers to realize higher yields with lower input costs. Previously, soybean genetic resources have been extensively studied for variation in soybean shoot architecture traits (e.g. plant height, node number and internode length). However, information on genetic and environmental impacts on soybean shoot architecture remain largely unknown. In order to address this issue, a recombinant inbred line (RIL) population containing 168 F_9_ lines was investigated for the presence of QTLs associated with three shoot architecture traits under four distinct environmental conditions. Further analysis was also conducted to identify correlations between QTLs and important environmental factors. The results presented here may contribute to efforts to breed soybean cultivars optimized for both shoot architectures and adaptation to diverse ranges in soil health properties.

## Results

### Effects of genotype × environment interactions on plant height traits in soybean

**Fig. 1:**
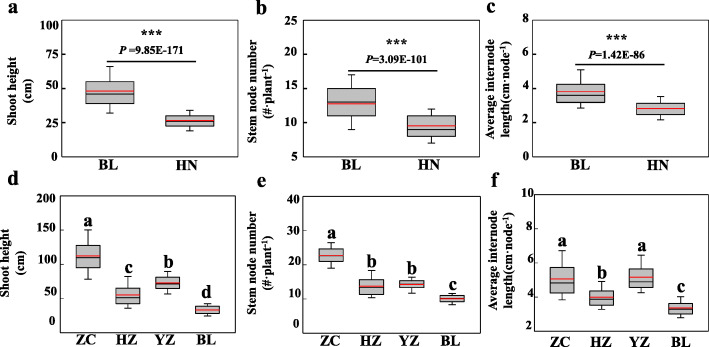
Plant height traits of soybean varied significantly among geographically distinct growth environments. **a-c** Plant height traits of 308 soybean cultivars selected from a core germplasm collection and grown in two distinct environments. **d-f** Plant height traits of 168 F_9_ recombinant inbred lines (RIL) grown in four environments. HN: Hainan, ZC: Zhao county, HZ: Hangzhou, YZ: Yangzhong, BL: Boluo; The black and red lines, lower and upper edges, and bars above or below the boxes represent median and mean values, 25th, 75th, 5th and 95th percentiles of all data, respectively; Asterisks and different letters over error bars indicate significant differences of the same trait among different environments in the Student’s t-test at 1‰ (*P*< 0.001) significance level

In order to evaluate whether G × E impact plant height in a natural soybean population, 308 representative cultivars from a core soybean germplasm collection [61] were selected and planted at two distinct experimental sites, Boluo (BL, 114.29°E, 23.17°N) and Hainan (HN, 109.48°E, 18.31°N). Three traits related to plant height (SH, shoot height; SNN, stem node number; and AIL, average internode length) were determined from field samples. In these tests, the mean values of SH, SNN and AIL were 81.46, 34.05 and 36.24% higher, respectively, in BL than that in HN (*P* value < 0.001) (Fig. 1a, b and c). This demonstrated that the plant height in soybean significantly varied between two distinct environments. Furthermore, genetic analysis suggested that the distributions for the three tested traits measured in two environments were approximately normal according to Kurtosis and Skewness values calculated over three replicates (Table 1). Broad-sense heritability (*h*^*2*^_*b*_) for all the traits under the tested environments varied from 0.74 to 0.92, with generally higher values being observed for SH than for the other two traits (Table 1). Regardless of these relatively small differences among traits, the results herein clearly suggest that variation in SH, SNN and AIL depend mainly on genotypic effects in a single environment. Across locations, however, values of *h*^*2*^_*b*_ for SH, SNN and AIL ranged between 0.38 and 0.40, all of which were significantly lower than in individual environments. Taken together, these results strongly suggest that SH, SNN and AIL are all greatly affected by both genotype and environment. In order to further determine G × E, two-way ANOVA was performed. As expected, the results showed that SH, SNN and AIL were significantly all affected (*P* value < 0.001) by environment, genotype and G × E (Table 2). However, the environment itself consists of many factors, including temperature, day-length, precipitation, soil properties and so on. To sort through these myriad environmental influences, we further evaluated the effects of several primary environmental factors, along with QTLs and QTL × environmental (QTL × E) on the tested traits. Analyzing specific environmental factors in this way might contribute to breeding soybean with shoot architectures optimized for specific sets of environmental conditions.

**Table 1:** Phenotypic variation and genetic analysis of plant height traits among 308 soybean germplasm varieties grown in two distinct environments

Trait ^**a**^	Env ^**b**^	Mean ± SD	MIN	MAX	CV%	Kurt	Skew	***h***^***2***^***b***
SH	BL	48.08 ± 14.68	12.00	110.00	30.52	2.42	1.14	0.92
	HN	26.50 ± 6.35	5.00	57.00	23.96	1.34	0.41	0.90
	Total	36.50 ± 15.4	5.00	110.00	42.20	2.37	1.29	0.39
SNN	BL	12.77 ± 3.01	3.00	24.00	23.60	0.27	0.27	0.89
	HN	9.53 ± 1.67	4.00	17.00	17.58	0.81	0.38	0.74
	Total	11.02 ± 2.88	3.00	24.00	26.13	0.81	0.85	0.40
AIL	BL	3.81 ± 0.94	1.23	10.00	24.77	3.90	1.41	0.81
	HN	2.80 ± 0.56	0.56	4.89	19.94	0.87	0.17	0.83
	Total	3.27 ± 0.91	0.56	10.00	27.96	4.06	1.34	0.38

Note: ^a^ SH, shoot height, cm; SNN, stem node number, #·plant^− 1^; AIL, average internode length, cm·per node^− 1^; ^b^ BL and HN represented the experimental sites of Boluo and Hainan, respectively; Total, includes data from both environments; MIN and MAX, the minimum and maximum values, respectively, of plant height traits; CV%, coefficient of variation; Kurt and Skew represent the Kurtosis and Skewness of plant height traits, respectively; *h*^*2*^*b*, broad-sense heritability

**Table 2:** ANOVA for variation of plant height traits among 308 soybean germplasm varieties grown in two distinct environments

Trait	***F*** values		
	Environment	Genotype	Environment × Genotype
SH	3420.19***	17.70***	17.67***
SNN	948.83***	15.80***	14.97***
AIL	942.26***	14.93***	12.05***

Note: *SH* shoot height, *SNN* stem node number, *AIL* average internode length; *** indicates significant differences at the 1‰ level (*P*< 0.001)

### Phenotypic variation among recombinant inbred lines

**Fig. 2:**
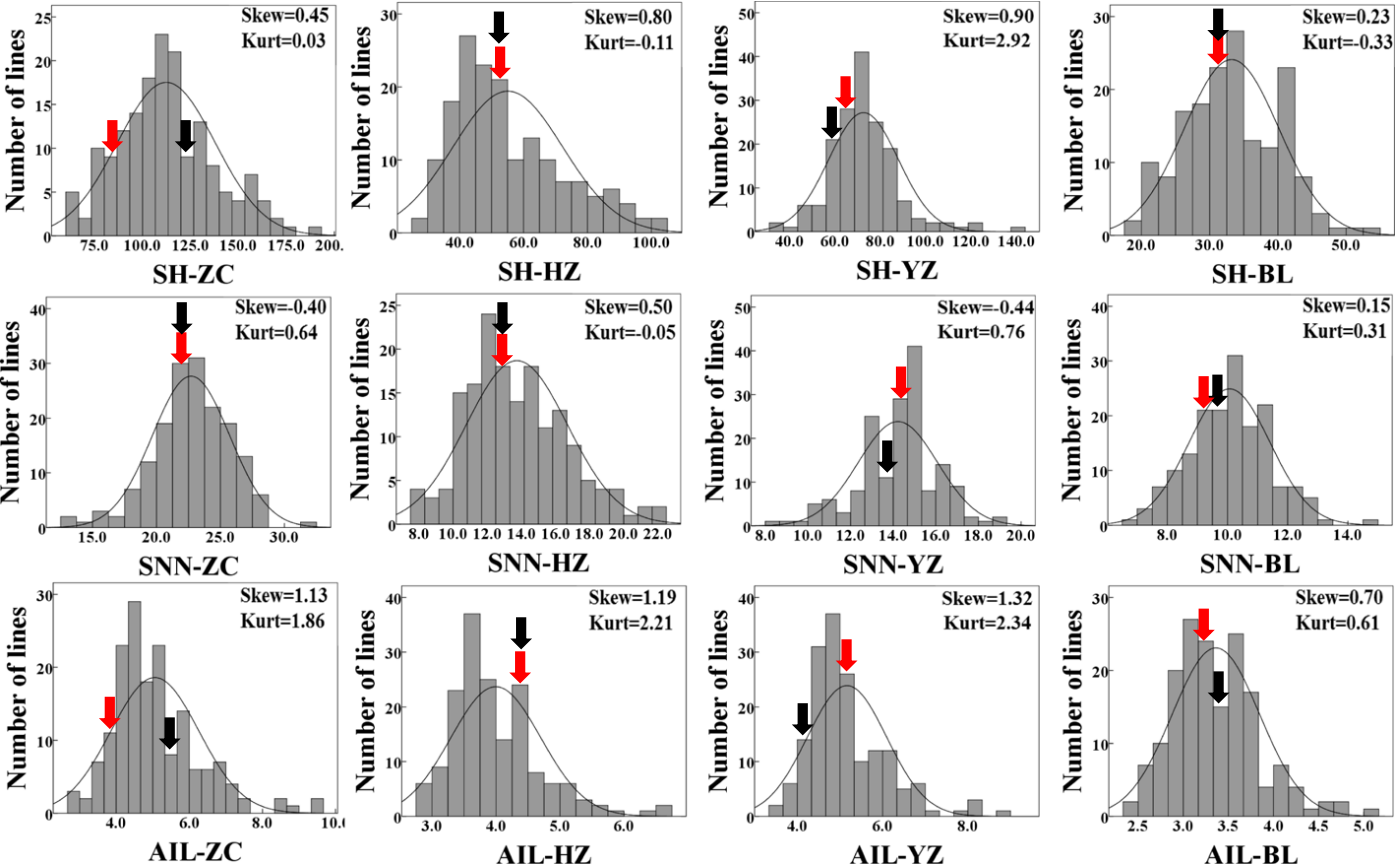
Distributions of plant height traits in 168 F_9_ RILs reared in four geographically distinct growth environments. Parental values are indicated by red (BX10) and black (BD2) arrows, respectively; Skew: Skewness; Kurt: Kurtosis; SH: shoot height; SNN: stem node number; AIL: average internode length; ZC: Zhao county, HZ: Hangzhou, YZ: Yangzhong, BL: Boluo

Given the prevalence of G × E identified for soybean in the plant height experiments above, two representative soybean accessions were, therefore, selected for developing a RIL population designed to explore QTL × E more fully in soybean. In addition, field characterizations were performed in an expanded set of four geographically distinct growth environments. In these trials, plant height traits of the parental lines, BX10 with the genotype of *E1E2E3E4E9dt1dt2tof11Tof12J* and BD2 with the genotype of *E1E2E3E4E9Dt1dt2Tof11tof12J*, significantly varied across the four tested environments, with observed ranges falling between 33.56 and 122.00 for SH, 9.63 and 23.00 for SNN, and 3.43 and 5.27 for AIL (Table 3). Although there were no significant differences observed between parental lines within individual environments, data from the RIL population exhibited maximum and minimum values beyond the parental extremes, and most of the distributions for traits tested across four environments were approximately normal according to Kurtosis and Skewness values calculated over three replicates (Fig. 2). These results suggest that soybean plant height traits are typical quantitative traits and both parents contain one or more genes contributing additively towards the tested traits. When sites were observed separately, the mean values of SH, SNN and AIL significantly varied in the ranges of 33.20–112.39, 10.07–22.70 and 3.36–5.06, respectively (Fig. 1d, e, f and Table 3)**,** implying large impacts of environmental factors on the tested traits. Furthermore, ANOVA results revealed that the variation observed for SH, SNN and AIL among RILs was significantly affected by environment and genotype, individually or in interaction terms (*P* value < 0.001) (Table 4). This was consistent with the results obtained from using the core collection germplasm cultivars (Table 2). Overall, the results herein demonstrate that the observed RIL population was suitable for further analysis.

**Table 3:** Phenotypic variation and genetic analysis of plant height traits among 168 F_9_ soybean RILs grown in four distinct environments

Trait ^**a**^	Env ^**b**^	Parents		RILs ^**c**^				
		BX10 ± SD	BD2 ± SD	Mean ± SD	MIN	MAX	CV%	***h***^***2***^***b***
SH	ZC	85.00 ± 5.29	122.00 ± 12.70	112.39 ± 25.51	63.33	191.67	22.70	0.82
	HZ	54.33 ± 4.62	54.67 ± 0.58	55.07 ± 17.15	29.33	102.00	31.14	0.91
	YZ	65.33 ± 4.62	61.00 ± 7.37	72.70 ± 15.32	35.00	141.33	21.08	0.84
	BL	33.56 ± 4.48	36.56 ± 6.67	33.20 ± 6.95	19.11	53.33	20.94	0.93
	Total	50.94 ± 20.53	57.83 ± 32.67	68.35 ± 33.94	19.11	191.67	49.65	0.76
SNN	ZC	22.33 ± 1.53	23.00 ± 1.41	22.70 ± 3.03	13.33	31.33	13.34	0.62
	HZ	12.33 ± 1.53	12.33 ± 1.53	13.73 ± 2.97	7.67	22.00	21.65	0.89
	YZ	13.00 ± 1.00	14.00 ± 2.65	14.22 ± 1.87	8.33	19.33	13.12	0.67
	BL	9.63 ± 1.30	10.11 ± 2.32	10.07 ± 1.35	6.78	14.89	13.37	0.77
	Total	12.94 ± 4.85	12.71 ± 4.62	15.18 ± 5.23	6.78	31.33	34.41	0.72
AIL	ZC	3.81 ± 0.14	5.27 ± 1.03	5.06 ± 1.20	2.69	9.51	23.76	0.72
	HZ	4.45 ± 0.24	4.45 ± 0.49	4.00 ± 0.70	2.75	6.74	17.59	0.70
	YZ	5.05 ± 0.55	4.37 ± 0.44	5.16 ± 0.93	3.50	8.81	18.00	0.67
	BL	3.43 ± 0.34	3.73 ± 0.89	3.36 ± 0.48	2.38	5.00	14.40	0.70
	Total	3.96 ± 0.71	4.15 ± 0.89	4.39 ± 1.15	2.38	9.51	26.16	0.75

Note: ^a^ SH, shoot height, cm; SNN, stem node number, #·plant^−1^; AIL, average internode length, cm·per node^−1^; ^b^ ZC, HZ, YZ, and BL represent the experimental sites of Zhao county, Hangzhou, Yangzhong and Boluo, respectively; Total, all the four environments combined; ^c^ RILs, recombinant inbred lines; MIN and MAX, the minimum and maximum values of plant height traits; CV%, coefficient of variation; *h*^*2*^*b*, broad-sense heritability

**Table 4:** ANOVA for variation in plant height traits among 168 F_9_ soybean RILs grown in four distinct environments

Trait	***F*** values		
	Environment	Genotype	Environment × Genotype
SH	5100.59***	15.15***	5.59***
SNN	2223.34***	4.46***	2.62***
AIL	585.38***	5.35***	2.23***

Note: *SH* shoot height, *SNN* stem node number, *AIL* average internode length; *** indicates significant differences at the 1‰ level (*P*< 0.001)

### Identification of QTLs contributing to plant height traits

A high-density genetic linkage map consisting of 3319 recombinant bin markers had been constructed using the RIL population developed in a previous study [62]. In order to identify significant QTLs, trait mean values were calculated for each RIL line. Subsequent QTL analysis identified a total of 19 significant loci containing 51 QTLs for the three tested traits, with 23, 13 and 15 QTLs being associated with SH, SNN and AIL, respectively. The LOD values of these QTLs ranged from 2.50 to 16.46, and explained 2.80–26.10% of phenotypic variation (Additional file 1: Table S1). Within environments, 13, 16, 13 and 9 QTLs were identified at the Zhao County (ZC, 114.48°E, 37.50°N), Hangzhou (HZ, 120.69°E, 30.51°N), Yangzhong (YZ, 118.20°E, 26.17°N) and BL field sites, respectively. However, only two loci, *Loc11* and *Loc19–1*, containing a total of 20 QTLs, were identified in each of the four distinct environments. Interestingly, the additive effect of *Loc11* was derived from BX10 and BD2 as determined in the two southern (including YZ and BL) and two northern (ZC and HZ) experimental stations, respectively. In addition, seven loci (QTLs) were significant only for single trait observed within one of the four tested environments. Other loci contributed to variation in two or more traits and/or at least two environments (Additional file 1: Table S1). The variation in significant QTL numbers and the extent of the additive effects of these QTLs suggests that soybean height QTLs might depend in part on specific environmental conditions present within individual sites, resulting in plant height influenced by genotype, environment, and G × E.

### QTL contributions to soybean plant height traits under varied environmental conditions

**Fig. 3:**
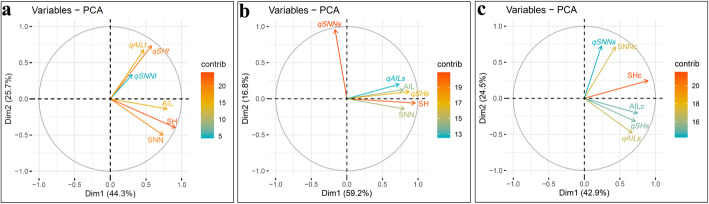
Principal component analysis (PCA) among detectable QTLs and soybean plant height traits under varied environments. The PCA plots were drawn based on **a** the three tested traits and total additive effects of QTLs for each trait; **b** the three tested traits and additive effects of QTLs in single environments, and **c** additive effects of QTLs in single environments and corrected values for each tested trait; SH: shoot height; SNN: stem node number; AIL: average internode length; *qSHt*, *qSNNt* and *qAILt* represent the sum of additive effects of QTLs for SH, SNN and AIL under all environments, respectively; *qSHs*, *qSNNs* and *qAILs* represented the sum of additive effects of QTLs for SH, SNN and AIL in single environments, respectively; SHc, SNNc and AILc represent corrected values for soybean SH, SNN and AIL, respectively; The contributions to phenotypic variation are represented by the color and length of vectors

In order to explore the stability of detected QTL contributions to plant height traits, QTL and plant height data from the four tested environments were subjected to principal components analysis (PCA). In this case, the first two principal components accounted for 44.3 and 25.7% of the total trait variation and QTL additive effects, respectively (Fig. 3a). Traits associated with plant height (SH, SNN and AIL) tended to group together, indicating a high correlation among them. In contrast, the total additive QTL effects for plant height traits (i.e. *qSHt*, *qSNNt* and *qAILt*) tended to group separately, to the extent that nearly 90° angles were observed among the directional vectors (Fig. 3a), which is indicative of these effects acting independently. These results suggest that the detected QTLs do not fully explain the extent of variation in plant height traits observed across varied environments, with the fact that most of these 51 QTLs were not significant in one or more tests reinforcing the conclusion that site specific conditions significantly influenced soybean height outcomes. To test this hypothesis, *qSHt*, *qSNNt* and *qAILt* were replaced by total additive QTL effects (*qSHs*, *qSNNs* and *qAILs*) from the corresponding environments in further PCA. Consistent with the previous PCA results, the first two principal components in this test accounted for 59.2 and 16.8% of the total variation, respectively (Fig. 3b). Besides the vector for *qSNNs*, the other 5 vectors grouped closely together (Fig. 3b), which suggests, consistent with our hypothesis, that the studied traits are highly correlated. On the other hand, the unexpected PCA results for *qSNNs*, the vector of which deviated considerably from the vector for SNN, strongly implied that environment differences greatly affected the QTLs for SNN. To minimize environment effects, plant height trait data (SH, SNN and AIL) were replaced by corrected data (SHc, SNNc and AILc) and subjected to PCA again. As expected, the first two principal components accounted for most of the variation, in this case, 42.9 and 24.5% of total variation, respectively (Fig. 3c). Additionally, all three vectors of additive effects (*qSHs*, *qSNNs* and *qAILs*) were relatively close to their corresponding traits (SHc, SNNc and AILc). Taken together, all of the results above strongly indicate that both G × E and QTL × E contribute to plant height phenotypes in the tested soybean population.

### Genotype × environmental factor interaction effects on plant height traits expressed in RILs

**Fig. 4:**
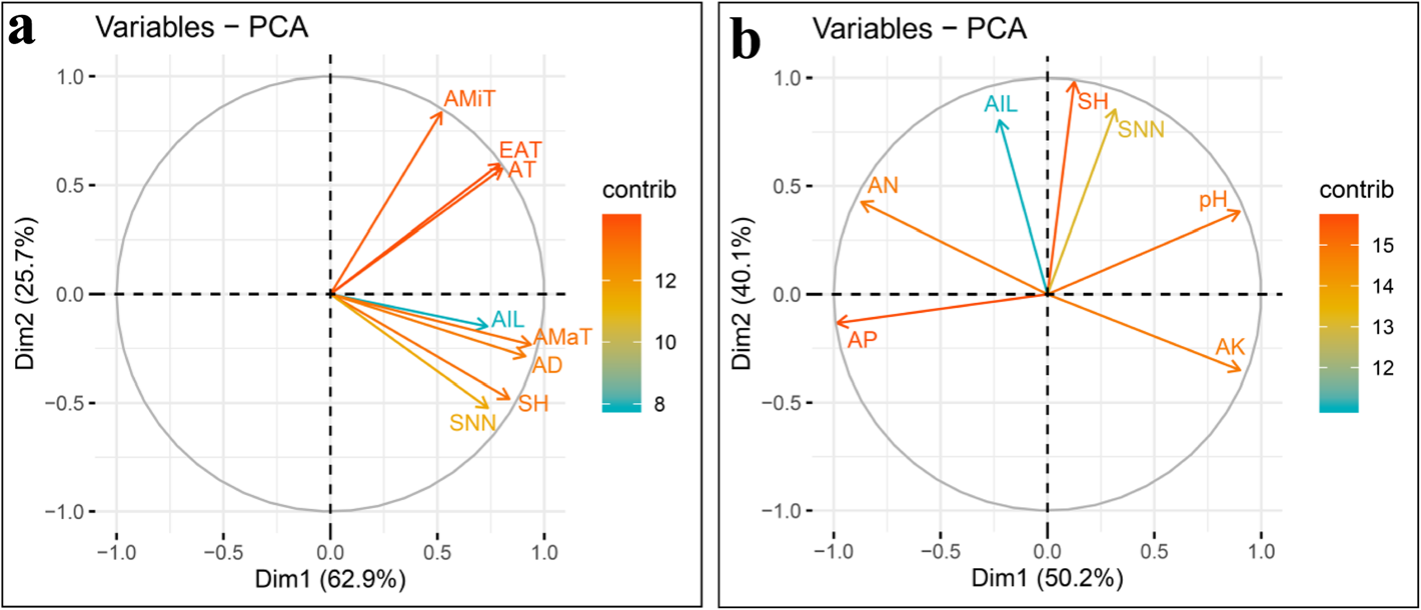
Principal component analysis (PCA) plot of relationships among plant height traits, agro-meteorological data and basic soil chemical properties. The PCA plots were drawn based on **a** the three plant height traits and agro-meteorological data, and **b** the three plant height traits and basic soil characteristics; SH: shoot height; SNN: stem node number; AIL: average internode length; AMaT: average maximum temperature; AMiT: average minimum temperature; AT: accumulated temperature; EAT: effective accumulated temperature; AD: average day-length; AN: available nitrogen; AP: available phosphorus; AK: available potassium; The contributions to phenotypic variation are represented by the color and lengths of the vectors

In order to further evaluate the effects of the main environmental factors on soybean plant height traits, correlation analysis and PCA were conducted with data collected for the tested traits, agro-meteorological factors and basic soil chemical properties. Results from PCA clearly showed that the first two principal components accounted for more than 88% of the total variation, and the vectors of AD and AMaT grouped closely with the vectors of SH, AIL and SNN (Fig. 4a). This suggests that both AD and AMaT contribute to enhance SH, SNN and AIL. Although, AMiT, EAT and AT grouped separately from most of the other vectors, their placement below 90°, implies that these three environmental factors might also enhance SH, SNN and AIL (Fig. 4a). This was further supported by the results from Pearson correlation analysis, in which significant correlations were identified among tested traits and agro-meteorological factors and correlation coefficients varied between 0.220–0.827 (*P* value < 0.01) (Table 5). Contrasting results were obtained when no vectors for soil factors grouped closely with SH, SNN or AIL (Fig. 4b). Except for the angle between pH and AN, all other angles between the AP and AK vectors and plant height traits were larger than 90°, which suggests that there were positive or negative interaction effects of pH and AN, or AP and AK on plant height traits (Fig. 4b). This was further confirmed in Pearson correlation analysis, in which significant positive correlations were established for pH and AN, and negative correlations for AP and AK with SH, SNN and AIL (Table 5). These results strongly demonstrate that both agro-meteorological and soil properties influence plant height traits, but the agro-meteorological factors largely predominate.

**Table 5:** Pearson correlation coefficients (r) for relationships among soybean plant height traits, agro-meteorological data, basic soil chemical properties, and additive effects of QTLs in individual environments

	SH	SNN	AIL	***qSHs***	***qSNNs***	***qAILs***
**AMaT**	0.827**	0.798**	0.602**	0.613**	−0.263**	0.483**
**AMiT**	ns	ns	0.220**	ns	0.491**	0.252**
**AT**	0.369**	0.267**	0.472**	0.207**	0.237**	0.411**
**EAT**	0.368**	0.297**	0.424**	0.244**	0.295**	0.399**
**AD**	0.821**	0.780**	0.613**	0.591**	−0.318**	0.475**
**pH**	0.461**	0.595**	0.092*	0.517**	ns	0.176*
**AN**	0.280**	0.115**	0.463**	ns	−0.203**	0.279**
**AP**	−0.250**	− 0.415**	0.109**	−0.382**	ns	ns
**AK**	−0.226**	−0.079*	− 0.374**	ns	0.349**	−0.182**

Note: *SH* shoot height, *SNN* stem node number, *AIL* average internode length, *AMaT* average maximum temperature, *AMiT* average minimum temperature, *AT* accumulated temperature, *EAT* effective accumulated temperature, *AD* average day-length, *AN* available nitrogen, *AP* available phosphorus, *AK* available potassium; *qSHs, qSNNs* and *qAILs* represent the sum of additive effects of QTL for soybean on shoot height, stem node number and average internode length in single environments; * and ** indicate significant correlations at the 5% (*P*< 0.05) and 1% (*P*< 0.01) levels, respectively

### QTL × environmental factor interactions in RILs

**Fig. 5:**
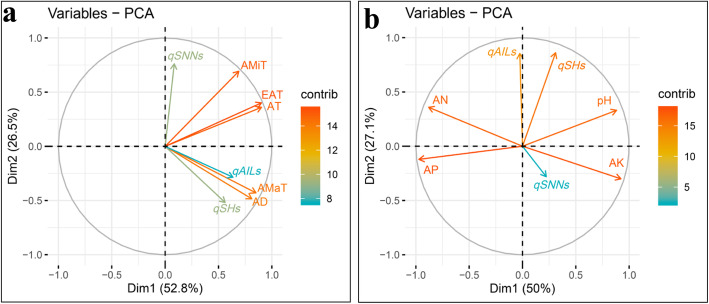
Principal component analysis (PCA) plots of relationships among detectable QTLs, agro-meteorological data and basic soil chemical properties. PCA plots were drawn based on relationships between **a** additive effects of QTLs in single environments and agro-meteorological data, and **b** additive effects of QTLs in single environments and basic soil characteristics; AMaT: average maximum temperature; AMiT: average minimum temperature; AT: accumulated temperature; EAT: effective accumulated temperature; AD: average day-length; AN: available nitrogen; AP: available phosphorus; AK: available potassium; *qSHs*, *qSNNs* and *qAILs* represent the sum of additive effects of QTLs on soybean shoot height, stem node number and average internode length, respectively, in single environment trials. The contributions to phenotypic variation are represented by the color and lengths of the vectors

In order to further explore the main factors imparting QTL additive effects, Pearson correlation analysis and PCA were also performed for agro-meteorological factors, soil properties and QTLs additive effects. Here, AD and AMaT closely grouped with *qSHs* and *qAILs*, while, AMiT, EAT and AT distributed separately (Fig. 5a), which is consistent with the relationships obtained in PCA of environmental factors and plant height traits (Fig. 4a). Interestingly, *qSNNs* aligned very closely with AMiT, yet were far from AMaT, suggesting that the additive effects of *qSNNs* increased with either increases in AMiT or reductions in AMaT. The positive relationship between *qSNNs* and AMiT, as well as, the negative relationship between *qSNNs* and AMaT were further confirmed by correlation analysis, in which the Pearson correlation coefficient was 0.491 between *qSNNs* and AMiT, or − 0.263 between *qSNNs* and AMaT (*P* value < 0.01) (Table 5). Further evaluation of soil properties and plant height traits showed that *qSHs* were significantly negatively correlated with AP, but positively correlated with pH. Meanwhile, *qSNNs* exhibited significant negative correlations with AN, and positive correlations with AK, while *qAILs* had significant positive correlations with two soil factors (pH and AN), but was negatively correlated with AK (Fig. 5b, Table 5). Taken together, these results demonstrate that both agro-meteorological factors and soil properties can significantly affect the additive effects of QTLs in regulating soybean plant height.

## Discussion

In contrast to environmental factors, genetic factors can be easily predicted and manually designed through traditional or modern techniques, such as cross-breeding or genetic modification. Furthermore, once genetic factors have been established, further monitoring of markers is unnecessary. Therefore, mining favorable alleles of QTLs conferring development of ideal plant heights became one of the most economic strategies employed to promote crop yield. Over recent decades, many researchers have attempted to identify stable QTLs regulating soybean plant height under varied environments, with a subset of these efforts seeking to clone the underlying genes [63–71]. To date, more than 304 QTLs have been documented in Soybase (https://www.soybase.org), however, many of the reported effects could not be confirmed in different environments, or their additive effects declined considerably in different conditions [16, 63, 67]. This reinforces the point suggested herein that QTLs effects depend on the specific environment conditions present where the soybeans are being grown. Therefore, it is unsurprising that only 2 loci (20 QTLs) out of the identified 19 loci (51 QTLs) were detected across all of the four distinct environments (Additional file 1: Table S1), and that the 51 detected QTLs could not explain a majority of the phenotype variation observed among RILs grown in the 4 diverse environments (Fig. 3a). Unfortunately, these “environmental QTLs”, which might play critical roles under specific environmental conditions, have been typically neglected in previous studies, possible due to more attention being devoted to detecting QTLs that remain stable under varied environmental conditions. Meanwhile, every advantage have its disadvantage, some QTLs possibly be omitted by using only one method to detect, especially for minor QTLs. In order to detect more genetic loci regulating soybean plant height, two algorithms, MQM and ICIM, were employed in this study. Among the 51 QTLs, more than half QTLs could be simultaneously detected by two methods, whereas 8 and 15 QTLs could be only detected by ICIM and MQM, respectively. We speculated that these inconsistent QTLs mainly derived from different algorithms between MQM and ICIM. However, most of the major QTLs could simultaneously detected by two methods, especially for QTLs which clustered in *Loc11* and *Loc19–1* and these inconsistent QTLs could explained more genetic variation under specific environmental conditions.

Under a given environment, shoot architecture were considerably regulated by flowering, maturity and growth habit of the soybean plants, and some genes underlined well-known genetic locus, such as *E1* - *E9*, *J*, *Tof11*(*time of flowering 11*), *Tof12*(*time of flowering 12*), *Dt1*and *Dt2*, were cloned [32–34, 43–49, 72]. Therefore, to further evaluate the affection of these flowering, maturity and growth habit-relate gene on soybean shoot architecture in our RIL population, the genotype of BX10 and BD2 were analysis basing on our recently published re-sequence data [62]. As expected, the sequence of three flowering, maturity and growth habit-related genes, *Tof11*, *Tof12* and *Dt1*, displayed significant variation between BX10 (*tof11Tof12dt1*) and BD2 (*Tof11tof12Dt1*) which possible could cause phenotype variation. Moreover, *Tof11* and *Dt1* were just located in the two environment stable locus (***Loc11*** and ***Loc19–1*)** which strongly that *Tof11* and *Dt1* underlying ***Loc11*** and ***Loc19–1***, respectively. Interestingly, the additive effect of *Loc11* was derived from BX10 and BD2 as determined in the two southern (including YZ and BL) and two northern (ZC and HZ) experimental stations, respectively. We assumed that this possibly due to the genetic roles of *Tof11* relied on photoperiod central gene, *E1*. Under short-day environments, the expression of *E1* was greatly suppressed [47, 72] which significantly impaired the function of *Tof11*, while the expression of two key *FT* homologs, *FT2a* (*FLOWERING LOCUS T*) and *FT5a* (*FLOWERING LOCUS T*), were significantly increased that leading to an earlier time of flowering and maturity and a relatively lower soybean plant height. Contrastingly, under long-day environments, dominant *Tof11* gene could significant enhance the expression of *E1* whereas significantly impair *FT2a* and *FT5a* expression which resulting in a later time of flowering and maturity and a relatively higher soybean plant height. Therefore, in our study, the contrasted functions of *Loc11* in low and high latitudes were largely dependent on the expression of its central gene, *E1* [72]. In addition, *Dt1*, which was just located in *Loc19–1*, is the most well-known gene of growth habit and plant height in soybean [33] and high expression of *FT5a* could accelerate terminating apical stem growth through inhabiting *Dt1* expression in post-flowering stage [73], which strongly implied that *Dt1* functions also relied on the expression of *E1*. Therefore, it is not surprised that the additive effect of *Loc19–1* was higher in long-day environments than that in short-day environments (Additional file 1: Table S1). Whatever, exploring and incorporating environmental factors that can regulate effective QTLs into breeding efforts should facilitate the development of new cultivars selected through marker assistant selection (MAS) that are adapted to produce grains in wide ranges of environmental conditions.

In order to facilitate the development of such breeding programs, various ecological environments have been classified and characterized throughout the main soybean producing countries [74–78]. For example, photoperiod and temperature are critical environmental factors that influence soybean shoot architecture development [38–40, 79–82]. In soybean, the effect of photoperiod on a variety of developmental processes has been well described, and more than 10 genetic loci sensitive to photoperiod changes have been cloned [40–42, 44, 83]. The sensitive alleles of these loci may enhance the duration of the soybean juvenile phase under long-day conditions, which leads to taller plants. Moreover, these photoperiod sensitive alleles have also been shown to play critical roles in the process of domestication and improvement, due of their ability to alter shoot architecture and enhance grain yields [44].

In contrast to the number of genes known to be photoperiod sensitive, temperature effects, though well documented, have not yet been adequately explained, and genetic loci sensitive to temperature remain rare. In this study, in order to explain the effects of temperature on soybean plant height, four temperature factors and three plant height traits were observed along with day-length. Interestingly, AMaT appeared to exert influence over the three tested plant height traits, whereas, AMiT, EAT and AT exhibited relatively small impacts (Fig. 4a). In addition, AMaT also affected AIL more than AD (Fig. 4a), which led to considerable impacts of AMaT on the QTLs of AIL (Fig. 5a). On the other hand, while the vector of SNN in PCA grouped with the vectors of SH and AIL (Fig. 4a), the vector of *qSNNs* was very distinct from those of *qAILs* and *qSHs* (Fig. 5a) which seemed that *qSNNs* did not significantly affect stem node number across environment. In this RIL population, both genotype of *E1*, the central gene of photoperiod [44, 72], were consistence in two parents. Recently, it was reported that *GmFT5a* and *GmAP1s* (*APETALA1*) could effectively terminate post-flowering stem node number [73, 84]. Then, under long day condition, high expression of *E1* could considerably inhibit *GmFT5a* and *GmAP1s* expression and significantly increase stem node number [47, 73, 84]. Therefore, the major variation of SNN across environments might be aroused by *E1* or *E1*-depended gene × environment interactions. Whatever, these results strongly indicate that variation of SNN across the tested environments is mainly regulated by G × E, but not QTL × E.

Higher temperatures are known to facilitate soybean node development. For instance, soybean node numbers increased from 18 to 29 and to 40 per plant when the temperature was increased from 30/22 °C to 38/30 °C and to 42/34 °C day/night regimes, respectively [85]. It has also been reported that the number of main stem nodes, plant height and mean internode length of crops increases with increasing temperature [86, 87]. However, no research has yet been conducted to determine the effects of diurnal temperature changes on soybean. For soybean, regions with large diurnal variations in temperature, such as Xinjiang Province in China, typically produce higher soybean yields [88]. In this study, we found that AMiT had a positive impact, and AMaT had a negative impact on enhancing the additive effects of QTLs for SNN. This might help to explain why large fluctuations in diurnal temperature can be beneficial for increasing soybean yield, though further work is needed to reveal the underlying molecular and genetic mechanisms.

Based on the present results, soil pH values appear to exert extensive influence over plant height (Fig. 5b and Table 5), possibly due to the fact that soils with low pH values offer limited bioavailability of N and P. The significant positive correlations were all established for pH and SH, SNN or AIL, as well as pH and *qSH* or *qAIL*, except for *qSNN* according to the PCA and Pearson correlation analysis (Figs. 4b, 5b and Table 5). Indicating that soybean plant height were increased by appropriately increasing the soil pH. So the additive effect of QTL related to soybean plant height traits might were promoted in ZC and HZ experimental stations and suppressed in YZ and BL experimental stations, which caused the additive effect of QTL were derived from BD2 detected in ZC and HZ, and the additive effect of QTL were derived from BX10 detected in YZ and BL. Therefore, on acid soils, fertilizers that can increase soil pH values should be first considered. In contrast, alkaline soils tend to have better nutrient availability conditions, and higher biological nitrogen fixation (BNF) capacities for soybean than their acidic counterparts. Over 70% of the N required for soybean growth can be derived from BNF [89], and excess N fertilizer input not only impairs the BNF capacity for soybean [62], but also leads to taller plants (Fig. 4b), which leads to poor lodging resistance. In addition, long-term fertilization with excessive amounts of N causes soil acidification [90–92], which often leads to deteriorating soil conditions. Therefore, in regions harboring alkaline soils, the amount of N fertilizers should be strictly controlled. Contrasted to K, fertilizers rich in P possible enhance AIL and decline SNN which unfavorable for final yield. However, P, which is critical for flower number, poding and filling grain [93, 94], is easily fixed by soil particles [95] or quickly leached out into water supplies, especially in acid soil of southern field and inadequate P may cause more serious yield lost. Therefore, fertilizers both rich in P and K should be considered for more extensive application.

## Conclusions

On the whole, the present study provides comprehensive results that contribute to understanding the relationships among environment, genotype, QTLs and soybean shoot architecture. Most importantly, these results also suggest that shoot architecture can be regulated not only by genetic modulators, but also by management strategies designed to optimize soil properties for soybean production. As such, this research opens new avenues for formulating strategies to breed soybean cultivars with improved shoot architectures geared towards sustainable production of high soybean yields in diverse environments.

## Methods

### Plant materials

A total of 308 representative soybean cultivars selected from an applied core germplasm collection [61] were included along with 168 F_9_ RIL progeny in tests for interactions between genotype and environment in effects on plant height traits, including shoot height (SH), stem node number (SNN) and average internode length (AIL). Experiments were conducted in geographically distinct field environments. Two cultivars, BX10 and BD2 with contrasted phenotype in flowering, photoperiod sensitivity, shoot architecture, and adaption ability in acidic soils, were selected to construct the RIL population using the single seed descent (SSD) method [96]. This RIL population was used to construct a genetic linkage map of QTLs for soybean plant height traits, as well as, to explore the genetic mechanisms underlying QTL × E. In addition, basing on analysis of re-sequence data [61], the genotype of some well-known flowering, maturity, and growth habit-related genes in BX10 and BD2 were *E1E2E3E4E9****dt1****dt2****tof11Tof12****J* and *E1E2E3E4E9****Dt1****dt2****Tof11tof12****J*, respectively.

### Field trials

The 308 soybean germplasm selections were planted in Boluo (BL, Guangdong province 114.29°E, 23.17°N), in 2018, and at the Hainan (HN, Hainan province 109.48°E, 18.31°N) experimental station in 2019. The 168 RIL progeny were grown at four experimental stations differing in agro-meteorological conditions and basic soil properties (Table 6). Specifically, these sites included the Zhao County (ZC, Hebei province 114.48°E, 37.50°N) experimental farm of the Institute of Genetics and Developmental Biology, Chinese Academy of Sciences, the Hangzhou (HZ, Zhejiang province 120.69°E, 30.51°N) experimental farm of the Institute of Crop and Nuclear Technology Utilization, Zhejiang Academy of Agricultural Sciences, BL and the Yangzhong (YZ, Fujian province 118.20°E, 26.17°N) experimental station of Fujian Agriculture and Forestry University. All of the trials were laid out as randomized complete block designs with three replications. Thirty seeds of each genotype were sown per plot in single 3 m rows spaced 0.5 m apart. None of the experiments were fertilized during soybean growth, and all of them incorporated consistent field management practices.

**Table 6:** Agro-meteorological data and basic soil chemical properties of the experimental locations

Experimental Locations		Agro-meteorological data					Soil basic chemical properties			
		AMaT	AMiT	AT	EAT	AD	pH	AN	AP	AK
ZC	114.48°E, 37.50°N	31.16	20.03	2353.50	1473.50	13.80	8.12	90.25	16.17	89.53
HZ	120.69°E, 30.51°N	29.31	21.56	2515.50	1635.50	12.98	7.85	65.56	14.74	132.42
YZ	118.20°E, 26.17°N	30.28	21.18	2701.50	1651.50	13.50	5.47	129.91	153.17	52.37
BL	114.29°E, 23.17°N	28.13	19.39	1913.50	1133.50	12.65	5.77	78.02	88.89	93.66
HN	109.48°E, 18.31°N	28.15	21.41	2329.50	1389.50	11.11	6.99	69.62	24.74	234.98

Note: *ZC* Zhao county, *HZ* Hangzhou, *YZ* Yangzhong, *BL* Boluo, *HN* Hainan, *AMaT* average maximum temperature, °C, *AMiT* average minimum temperature, °C, *AT* accumulated temperature, °C·d, *EAT* effective accumulated temperature, °C·d, *AD* average day-length, hours·day^− 1^, *AN* available nitrogen, mg·kg^− 1^; *AP* available phosphorus, mg·kg^− 1^; *AK* available potassium, mg·kg^− 1^

### Plant sampling and genetic analysis

SH and SNN of three representative plants from each line were directly measured in the field at the R6 stage, and AIL was calculated as the ratio of SH to SNN. All the data were used to determine the effect of genotype and environment on the tested traits through Two-Way ANOVA in SPSS 19 [97], and to estimate the broad sense heritability of each trait in each or all environments using the formula *h*^*2*^*b* = VG/(VG + VE), with VG and VE as the respective variance between and within RILs.

### Measurements of agro-meteorological and basic soil chemical properties

The agro-meteorological data from each field site are listed in Table 6. Among measured conditions, maximum temperature (MaT, °C), minimum temperature (MiT, °C) and average day-length (AD, hours·day^− 1^) were obtained from weather data deposited at http://tianqi.2345.com/ and https://www.51240.com/. Average maximum temperature (AMaT, °C), average minimum temperature (AMiT, °C) and accumulated temperature (AT, °C·d) were calculated as:

$$ AMaT=\left(\sum \limits_{r=1}^n MaTr\right)/n $$

$$ AMiT=\left(\sum \limits_{r=1}^n MiTr\right)/n $$

$$ AT=\left(\sum \limits_{r=1}^n MaTr+\sum \limits_{r=1}^n MiTr\right)/2 $$

Meanwhile, due to effective accumulated temperature (EAT, °C·d), which mean the sum of the difference value between the daily average temperature and biological zero point, could significantly affects plant growth and development [98], the EAT for soybean during seeding to R6 stage were also evaluated. For soybean, the biological zero point is 10 °C and EAT for soybean was calculated as follows:

$$ EAT=\left[\sum \limits_{r=1}^n\left( MaTr-10\right)+\sum \limits_{r=1}^n\left( MiTr-10\right)\right]/2 $$

where *MaTr* and *MiTr* are the MaT and MiT of the *r*th (*r*=1, 2, …,*n*) day of soybean growth, respectively.

The basic soil chemical properties of the top 0–20 cm of soil at each location were determined using 10 randomly collected soil samples from each experimental site. The soil pH, available nitrogen (AN), available phosphorus (AP) and available potassium (AK) as measured according to soil and agricultural chemistry analysis protocols [99] are listed in Table 6.

### Genetic linkage map and QTL mapping

Based on a previously constructed genetic linkage map [62], the mean value of each trait from three plants in each plot was used to identify significant quantitative trait loci (QTL) using QTL IciMapping version 4.1 running the inclusive composite interval mapping (ICIM) method [100], as well as in MapQTL6.0 running interval mapping (IM) and Multiple-QTL model (MQM) algorithms [101]. For QTL IciMapping version 4.1, the mapping method of ICIM-ADD was selected to identify QTLs with the step width, probability in stepwise regression and LOD threshold being set to 1 cM, 0.001 and 2.5, respectively. First, IM analysis was conducted for QTL analysis, and the markers with the highest LOD scores were selected as cofactors to carry out MQM analysis. QTLs with LOD score exceeding 2.5 were considered as high confidence QTLs in MQM mapping. To precisely evaluate extensive affection of the candidate QTLs, separated QTLs which clustered together on linkage group were combined as a genetic locus.

### Evaluation of genetic effects on plant height parameters measured at four distinct locations

The sum of additive effects for QTLs identified in each single environment (*qSHs*, *qSNNs* and *qAILs*) or all four environments (*qSHt*, *qSNNt* and *qAILt*) were evaluated by principal component analysis (PCA). The *qSHs*, *qSNNs*, *qAILs*, *qSHt*, *qSNNt* and *qAILt* were calculated as follows:

$$ {qTs}_{ij}=\sum \limits_{r=1}^k{Ar}_{ij} $$

$$ {qTt}_j=\sum \limits_{r=1}^k{Ar}_j $$

$$ qTs=\left({qTs}_{i1},{qTs}_{i2},\cdots, {qTs}_{ij}\right) $$

$$ qTt=\left({qTt}_1,{qTt}_2,\cdots, {qTt}_j\right) $$

where *qTs*_*ij*_ and *qTt*_*j*_ are the total additive effects of QTLs for tested traits in the *j*th (*j*=1, 2, …, 168) RIL in single environment and combined environment trials, respectively. The other parameters are *Ar* representing the additive effect of the *r*th (*r*=1, 2, …, *k*) QTL, *qTs* representing the *qSHs*, *qSNNs* or *qAILs*, *qTt* representing *qSHt*, *qSNNt* or *qAILt*, and *i* signifying the experimental station (ZC, HZ, YZ and BL).

Environmental effects were eliminated from additive QTL effects, with the values of SH, SNN and AIL being corrected and named as SHc, SNNc and AILc, respectively. The formulas used are listed as follows:

$$ {Tc}_j={T}_j- Tm $$

$$ Tc=\left({Tc}_1,{Tc}_2,\cdots, {Tc}_j\right) $$

where *Tc*_*j*_ and *T*_*j*_ are the respective corrected  and measured  values of each trait for the *j*th (*j*=1, 2, …, 168) RIL, and *Tm* is the mean of 168 RILs for each trait in a single environment, and *Tc* represents the SHc, SNNc or AILc.

### PCA analysis

QTL × E effects on soybean plant height were assessed by PCA performed with the tested traits, agro-meteorological data, basic soil properties, additive effects of QTLs and corrected values for each trait using R with the packages ‘factoextra’, ‘factoMineR’ and ‘ggplot2’, along with the function ‘fviz_pca_var’ [102–104] (version 3.6.1, https://www.r-project.org/).

## Supplementary Information

**Additional file 1: Table S1.** Putative QTLs detected for plant height traits by MapQTL 6.0 and QTL IciMaping 4.1 using 168 F_9_ soybean RILs under different environments.

## Data Availability

The datasets generated and/or analysed during the current study are available in the National Centre for Biotechnology Information (NCBI) repository, and the accession number of BioProject Database and Sequence Read Archive (SRA) Database are PRJNA688599 and SRR13341345 - SRR13341514, respectively. All the supporting data are available from the corresponding author on reasonable request (yyq287346@163.com).

## Abbreviations

SH: Shoot height
SNN: Stem node number
AIL: Average internode length
SSD: Single seed descent
ZC: Zhao County
HZ: Hangzhou
YZ: Yangzhong
BL: Boluo
HN: Hainan
MaT: Maximum temperature
MiT: Minimum temperature
AD: Average day-length
AMaT: Average maximum temperature
AMiT: Average minimum temperature
AT: Accumulated temperature
EAT: Effective accumulated temperature
AN: Available nitrogen
AP: Available phosphorus
AK: Available potassium
QTL: Quantitative trait locus
*h*^*2*^*b*: Broad-sense heritability
*qSHs*: Sum of additive effects of QTLs for SH in single environments
*qSNNs*: Sum of additive effects of QTLs for SNN in single environments
*qAILs*: Sum of additive effects of QTLs for AIL in single environments
*qSHt*: Sum of additive effects of QTLs for SH under all environments
*qSNNt*: Sum of additive effects of QTLs for SNN under all environments
*qAILt*: Sum of additive effects of QTLs for AIL under all environments
SHc: Corrected values for soybean SH
SNNc: Corrected values for soybean SNN
AILc: Corrected values for soybean AIL
Env: Environment
SD: Standard deviation
MIN: Minimum values
MAX: Maximum values
CV%: Coefficient of variation
Kurt: Kurtosis
Skew: Skewness
PVE(%): Percentage of phenotypic variance explained by the QTL
Add: Additive effects
